# Zinc-Modified Mordenite Zeolite as a Molecular Carrier for Donepezil: A Framework for Drug Delivery Applications

**DOI:** 10.3390/molecules30214174

**Published:** 2025-10-24

**Authors:** Diana Guaya, Lupe Carolina Espinoza, Ximena Jaramillo-Fierro, Dagmar Gualotuña Campoverde, Lilian Sosa, Ana Cristina Calpena

**Affiliations:** 1 Departament of Chemistry, Universidad Técnica Particular de Loja, Loja 1101608, Ecuador; deguaya@utpl.edu.ec (D.G.); xvjaramillo@utpl.edu.ec (X.J.-F.); 2 Institute of Nanoscience and Nanotechnology (IN2UB), University of Barcelona, 08028 Barcelona, Spain; 3 Carrera de Bioquímica y Farmacia, Universidad Técnica Particular de Loja (UTPL), Calle París s/n y Praga, Loja 110107, Ecuador; degualotuna1@utpl.edu.ec; 4 Institute of Microbiology Research (IIM), Faculty of Sciences, National Autonomous University of Honduras (UNAH), Tegucigalpa 11101, Honduras; lilian.sosa@unah.edu.hn; 5 Pharmaceutical Technology Research Group, Faculty of Chemical Sciences and Pharmacy, National Autonomous University of Honduras (UNAH), Tegucigalpa 11101, Honduras; 6 Department of Pharmacy, Pharmaceutical Technology and Physical Chemistry, Faculty of Pharmacy and Food Sciences, University of Barcelona, 08028 Barcelona, Spain

**Keywords:** donepezil, zinc-modified mordenite, controlled release, drug carriers

## Abstract

The development of advanced drug delivery systems is essential for improving therapeutic efficacy, particularly in the treatment of neurodegenerative disorders such as Alzheimer’s disease. This study investigates zinc-modified mordenite zeolite (MR-ZN) as a novel platform for the controlled delivery of donepezil (DPZ), a cholinesterase inhibitor. Natural mordenite was modified with zinc, enhancing its surface area from 62.1 to 85.4 m^2^/g and improving its adsorption properties. Donepezil was successfully loaded at two doses (10 mg and 23 mg), achieving high loading efficiencies of 95% and 94%, respectively. Adsorption kinetics followed a pseudo-second-order model (R^2^ > 0.99), indicating that chemisorption predominates through coordination between DPZ functional groups and Zn^2+^ sites, while complementary physisorption via hydrogen bonding and van der Waals interactions also contributes to molecular stabilization within the zeolite framework. In vitro release studies under simulated gastrointestinal conditions demonstrated sustained and pH-responsive release profile with 80% and 82% of donepezil released after 24 h for 10 mg and 23 mg formulations, respectively. Density Functional Theory (DFT) calculations revealed favorable adsorption energy (−26.4 kJ/mol), while Bader and Electron Localization Function (ELF) analyses confirmed hydrogen bonding and electrostatic interactions without compromising the zeolite framework. These findings validate MR-ZN as structurally stable, efficient, cost-effective and biocompatible matrix for oral drug delivery. The combination of experimental data and theoretical modeling supports its potential to improve bioavailability and therapeutic performance in neurodegenerative treatment.

## 1. Introduction

Alzheimer’s disease (AD) represents a major global health challenge, characterized by progressive cognitive decline and memory impairment. Despite decades of intensive research and therapeutic development, no definitive cure has been found for this neurodegenerative disorder, and currently available treatments offer only limited symptomatic relief [1]. A critical challenge in AD treatment lies in the effective delivery of drugs to the brain, where they can exert their pharmacological effects while minimizing side effects and toxicity. The blood–brain barrier (BBB) constitutes a significant obstacle, restricting the passage of many therapeutic agents into the brain parenchyma [2]. Moreover, drugs that do cross the BBB often undergo rapid clearance or metabolic degradation, necessitating frequent dosing and increasing the risk of adverse effects [3]. As the global prevalence of AD continues to rise with aging population, there is an urgent need to develop innovative therapeutic strategies to mitigate its impact [4]. Among the current pharmacological options, donepezil has emerged as a prominent cholinesterase inhibitor, demonstrating beneficial effects on cognitive function and quality of life in AD patients [5]. To enhance its therapeutic efficiency and overcome the limitations of conventional administration, increasing attention has been directed toward the development of controlled and sustained drug delivery systems for donepezil [6].

In the pursuit of advanced drug delivery systems, the field of pharmaceutical sciences continually seeks novel materials and technologies to revolutionize formulation and efficacy. Zeolites, a class of microporous aluminosilicate minerals, have emerged as promising carriers for the controlled release of therapeutic agents in AD therapy [7]. The efficacy of zeolitic materials as drug carriers is closely related to their physicochemical characteristics, including high surface area, well-defined pore structure, ion-exchange capacity, and tunable surface acidity [8]. These features explain why zeolites, used in industrial processes such as gas separation, ion exchange, and catalysis [9]; are now being explored for pharmaceutical applications involving selective adsorption, controlled release, and chemical modification to enhance drug–carrier interactions and stability. Particularly, mordenite-type, zeolites have recently gained increasing attention in pharmaceutical research. Mordenite possesses advantageous molecular sieving properties, release control capabilities, and the ability to enhance solubility and bioavailability of poorly water-soluble drugs [10].

Zeolite-type mordenite is distinguished by its excellent thermal stability, biocompatibility, and high adsorption capacity, making it a promising candidate for a wide range of pharmaceutical applications [11]. Its well-defined porous structure and exceptional adsorption properties make it particularly suitable as a vehicle for donepezil delivery in Alzheimer’s disease treatment [12]. Encapsulating donepezil within the zinc-modified mordenite matrix aims to achieve sustained drug release, thereby enhancing therapeutic efficacy. This innovative approach has the potential to improve patient outcomes, increase donepezil bioavailability, and ultimately help mitigate the cognitive decline associated with AD [13]. The zinc-modified mordenite exhibits favorable characteristics such as structural homogeneity, enhanced thermal and chemical stability, and safety for human use. Furthermore, zinc plays an essential role in enzymatic catalysis and neuronal signaling [14]. Zinc ions within the zeolite structure not only enhance its stability but also provide mordenite the capacity to interact with key molecular targets implicated in AD pathology. It is well-established that zinc has the potential to reduce brain damage and improve neurological performance [15]. In this study, the incorporation of Zn^2+^ into the mordenite framework improves its surface reactivity and adsorption affinity toward donepezil through stronger coordination and electrostatic interactions, which are expected to favor a more controlled and sustained release behavior. One of the most compelling features of zinc zeolites in the context of AD treatment is their ability to maintain prolonged exposure of brain tissues to therapeutic agents. Given the chronic nature of AD, maintaining therapeutic drug levels over extended periods is essential [16].

Some studies have attempted to understand adsorption processes at the microscopic and macroscopic levels using statistical physics models. These models can predict adsorption mechanisms based on physical parameters; however, they could be complemented with quantum mechanical calculations based on Density Functional Theory (DFT) to clarify adsorption mechanisms at the molecular level [17]. Despite the importance of using computational simulation to understand the adsorption mechanisms of various materials, to date, no studies have reported the use of quantum computational methods to describe the adsorption mechanism of donepezil (DPZ) on mordenite zeolite (MR). Therefore, the computational results reported in this study will contribute to a better understanding of the interaction between donepezil and mordenite zeolite, thus clarifying the experimentally obtained results.

This work represents a contemporary research development, showcasing a key case study and discussing the prospects of zinc-modified mordenite-type zeolite in pharmaceutical sciences. This versatile material exemplifies the convergence of materials science and pharmaceutical innovation, holding the promise of enhancing the efficacy and safety of drug delivery systems designed for Alzheimer’s therapy. Through a comprehensive analysis of scientific literature and a critical evaluation of experimental data, this study aims to shed light on the potential of zinc zeolites to transform Alzheimer’s disease treatment strategies by providing a sustained and targeted drug delivery platform.

The specific aims of this study are to (a) perform molecular modeling using density functional theory (DFT) simulation to investigate the interactions mechanisms between donepezil and the mordenite framework, (b) synthesize and characterize natural and zinc-modified mordenite zeolites through techniques such as X-ray fluorescence (XRF), X-ray diffraction (XRD), scanning electron microscopy (SEM), and Fourier-transform infrared spectroscopy (FTIR); and (c) evaluate drug-loading efficiency and release behavior of donepezil from these zeolitic carriers under simulated gastrointestinal conditions, assessing their potential as controlled drug delivery systems. The experimental findings are integrated with computational modeling to provide a comprehensive understanding of the drug retention and release behavior, assessing the feasibility of using zinc-modified mordenite as a stable, safe, and efficient mineral-based drug delivery platform.

## 2. Results and Discussion

### 2.1. Theoretical Insights into Mordenite Structure and DPZ Adsorption Potential

**Figure 1:**
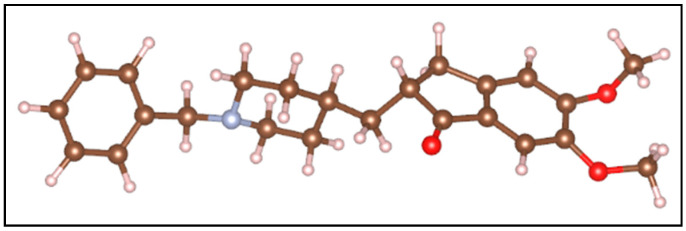
Optimized molecular structure of donepezil used in DFT simulations. The red spheres represent oxygen atoms, the brown spheres carbon atoms, the gray spheres nitrogen atoms, and the white spheres hydrogen atoms.

In this study, the adsorption of donepezil on mordenite zeolite was investigated using a density functional theory (DFT) approach. The DFT analysis was limited to mordenite, given its predominant abundance and larger pore dimensions compatible with the donepezil molecule. Consequently, adsorption studies were not extended to heulandite or clinoptilolite, whose main cavities are smaller than those of mordenite. Figure 1 presents the optimized molecular structure of the donepezil molecule, which was found to be 18.2 Å in length, 7.5 Å in height, and 6.2 Å in width.

For the mordenite zeolite, a realistic structural model was employed, consisting of 675 atoms, 802 bonds, and 192 polyhedral. This model provided an accurate representation of the complex interactions within the framework, thereby enhancing the reliability of the adsorption simulation [17]. The unit cell parameters and volume before and after full optimization of both pristine mordenite and the donepezil-loaded mordenite systems are summarized in Table 1.

**Figure 2:**
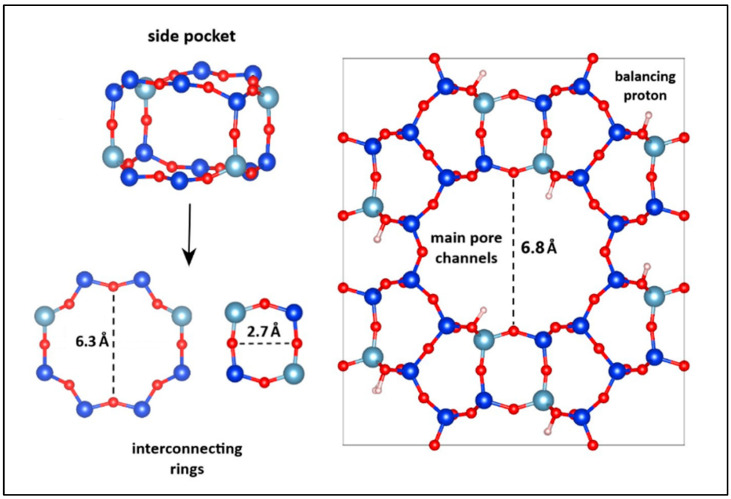
Structural model of the mordenite zeolite obtained using Materials Studio 2017. The blue spheres represent silicon atoms, the cyan spheres aluminum atoms, the red spheres oxygen atoms, and the white spheres hydrogen atoms. The dotted line indicates the distance between the two opposite points used to determine the pore size.

The resulting molecular formula of the mordenite zeolite was H_32_Si_160_Al_32_O_369_∙15H_2_O. The smallest distance between two opposing oxygen atoms in the main pore channels of MOR was found to be 6.8 Å. These primary channels are formed by twelve-membered oxygen rings, while adjacent side pockets are framed by rings composed of four to eighth oxygen atoms. The minimum diameters of the rings forming the large and small pockets were 6.3 Å and 2.7 Å, respectively. Figure 2 depicts the orthorhombic unit cell of the mordenite zeolite, as modeled using Materials Studio software.

**Figure 3:**
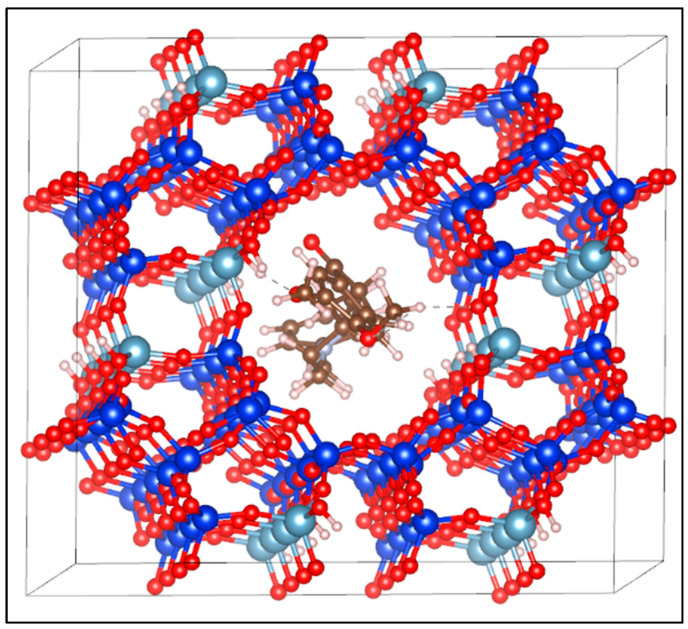
Structural model of the donepezil-loaded mordenite (MOR-DPZ) supersystem constructed using Materials Studio 2017. The blue spheres represent silicon atoms, the cyan spheres aluminum atoms, the red spheres oxygen atoms, the brown spheres carbon atoms, the gray spheres nitrogen atoms, and the white spheres hydrogen atoms. The dotted line indicates the distance between the two opposite points.

Adsorption energy was employed as a key criterion to describe the interaction between the donepezil molecule and the mordenite framework [4]. The calculated adsorption energy was 26.4 kJ/mol, indicating an exothermic interaction. This energy value is characteristic of moderate binding, suggesting that the adsorption of donepezil onto mordenite is primarily governed by physical forces and hydrogen bonding rather than strong covalent bonding. Despite the moderate interaction strength, the adsorption is sufficiently exothermic to stabilize the donepezil molecule at the adsorption site without compromising the reversibility of the process, an essential feature for controlled release applications. Following full optimization, the average hydrogen bond distance formed between mordenite and donepezil, specifically between H_(MOR)_-O_(DPZ)_, was approximately 1.8 Å. Figure 3 illustrates the donepezil-loaded mordenite supersystem, constructed using Materials Studio software, showing the drug molecule accommodated within the primary twelve-membered ring channels of the zeolite framework.

Regarding the donepezil molecule, it was observed that after adsorption into the large cavity of mordenite, its dimensions changed slightly due to structural reorganization. The molecule, initially measuring 18.2 Å in length, 7.5 Å in height, and 6.2 Å in width, adjusted to 18.1 Å in length, 8.0 Å in height, and 7.9 Å in width. This variation can be attributed to spatial confinement within the zeolite and the formation of hydrogen bonds between the molecules and the cavity walls. These interactions induce a slight compression along the molecular length and an expansion in height and width. The conformational flexibility of donepezil, combined with Van der Waals forces, facilitated this adjustment, allowing the molecule to maximize favorable interactions with the zeolite framework.

Furthermore, to verify the chemical interaction between mordenite and the donepezil, a Bader population analysis was performed on the donepezil-loaded mordenite systems [18,19]. This method is particularly valuable as it enables the description of bond ionicity by quantifying the charge transfer between bonded atoms [20]. Table 2 presents the average charge per atom type before (BA) and after (AA) donepezil adsorption. Atoms associated with the adsorbent surface are denoted by the subscript (_MOR_), whereas those belonging to donepezil are labeled (_DPZ_). Bader population analysis and electron localization function (ELF) evaluations provided detailed insights into the nature of the interactions between donepezil and mordenite. The observed charge transfer, evidenced by a reduction in electron density around the nitrogen atom of donepezil, supports the existence of electrostatic interactions with zeolite. This finding is consistent with an adsorption mechanism that, although not dominantly covalent, involves significant chemical interaction contributing to the stability of the adsorbate-adsorbent complex. The ELF analysis revealed electron density redistribution indicative of partially covalent interaction at specific contact points, although ion-dipole and dipole–dipole interactions predominate, which are typical of hydrogen bonds and Van der Waals forces. These results reinforce the hypothesis that donepezil adsorption onto mordenite occurs through a combination of interaction types, offering potential benefits for controlled drug release by balancing molecular stability with reversible desorption under appropriate conditions.

**Figure 4:**
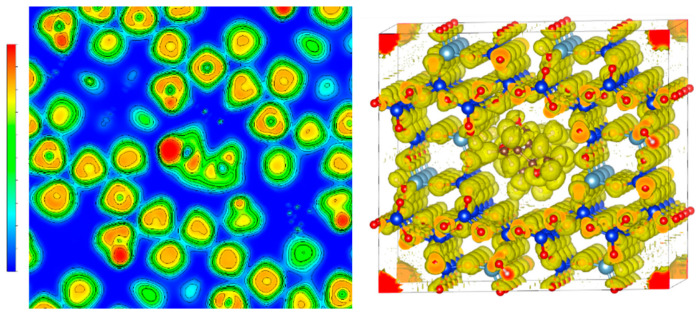
Electron localization function (ELF) analysis of donepezil-loaded mordenite showing electron density redistribution upon adsorption.

In addition, the Electron Localization Function (ELF) was employed to further elucidate the nature of interactions between mordenite (MOR) and donepezil (DPZ) [21,22]. ELF analysis provides insight into bonding character by examining the distribution of maximum electron density (RMD) around atomic nuclei. A symmetrical RMD distribution around the nucleus suggests ionic or van der Waals-type interactions, whereas covalent character increases as the RMD shifts between atomic centers, resulting in symmetrical distributions typical of ideal covalent bonds. The ELF depiction for the MOR-DPZ interaction, presented in Figure 4, indicates an enhanced level of interaction between the donepezil molecule and the mordenite framework. This visualization reveals RMDs aligned along the axis connecting atomic nuclei, suggesting electron sharing pathways. However, within the mordenite/donepezil complex, RMDs do not fully envelop the nuclei [23], implying partial covalency within localized regions.

The molecular modeling and DFT simulations complement the experimental results by illustrating the steric constraints and interaction energies governing donepezil adsorption. These findings highlight the limited penetration of the molecule into the narrower mordenite channels and the predominance of surface adsorption and defect sites as primary interaction domains. Thus, the simulations provide a mechanistic framework that supports and explains the adsorption and release behavior observed experimentally.

### 2.2. Experimental Characterization of Zinc-Modified Mordenite as a Drug Carrier

The chemical composition of the zeolitic samples (Table 3) demonstrates that the modification processes did not significantly alter the fundamental composition of the zeolite. The main components, such as silicon dioxide (SiO_2_) and aluminum oxide (Al_2_O_3_), along with trace elements, remained largely unaffected by the zinc modification. It is important to consider that some variations may originate from inherent impurities present in the natural zeolitic material, contributing to the observed variability in the chemical composition. The accuracy of the method used to determine the chemical composition is usually limited; however, it provides a useful indication of the changes occurring in the material during the modification and adsorption processes.

The Si/Al ratio, a critical parameter that defines the structural and functional properties of zeolite, was 4.5 in the MR sample and decreased to 4.2 following the modification process. This ratio governs the charge balance of the zeolitic framework and directly influences both ion exchange capacity and adsorption characteristics. This suggests the structural robustness of the mordenite framework, which retained its essential compositions even after undergoing ion exchange with zinc. Notably, the zinc oxide content appeared higher in the zinc-modified sample (MR-ZN) compared to the unmodified zeolite (MR). Figure S1 illustrates the speciation diagram of zinc acetate in aqueous solution as a function of pH, generated using the Hydra-Medusa equilibrium software (Supplementary Material). At strongly acidic conditions (pH < 4), the system is dominated by free Zn^2+^ ions and soluble acetate complexes such as Zn(CH_3_COO)^+^ and Zn(CH_3_COO)_2_, ensuring high availability of Zn^2+^ for cation exchange with alkali or alkaline earth cations (e.g., Na^+^, K^+^, Ca^2+^) present in the zeolite framework. This regime is particularly favorable for ion-exchange modification, as the zinc species remain soluble and can diffuse into the microporous structure of mordenite without precipitation. However, it is important to note that elements such as sodium, which are not detectable by XRF technique, may influence the reported percentage of other elements. Furthermore, the chemical analysis revealed interactions with other elements, including sodium (Na), potassium (K), and calcium (Ca), during the zinc modification process. While their concentrations exhibited only minor fluctuations, these elements play significant roles in the ion exchange process with zinc. According to the results of atomic absorption spectrometry, Na (8.5 mg·L^−1^, attributed to the NaOH used during the modification process), K (0.9 mg·L^−1^) and Ca (0.2 mg·L^−1^) were present in the zinc exhausted solution after zeolite modification, confirming that the higher charge density of divalent zinc favors substitution of monovalent ions (e.g., sodium and potassium), thereby better compensating the negative charge of aluminosilicate regions in the zeolite. In addition, the lower ionic radius of Zn^2+^ (0.74 Å) compared with Na (1.02 Å), K (1.38 Å), and Ca (1.00 Å) allows it to occupy more closely coordinated sites [24].

**Figure 5:**
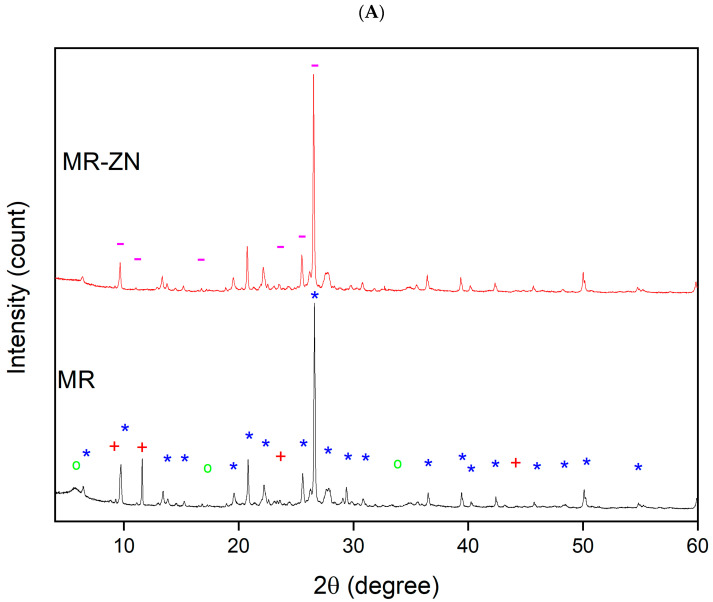

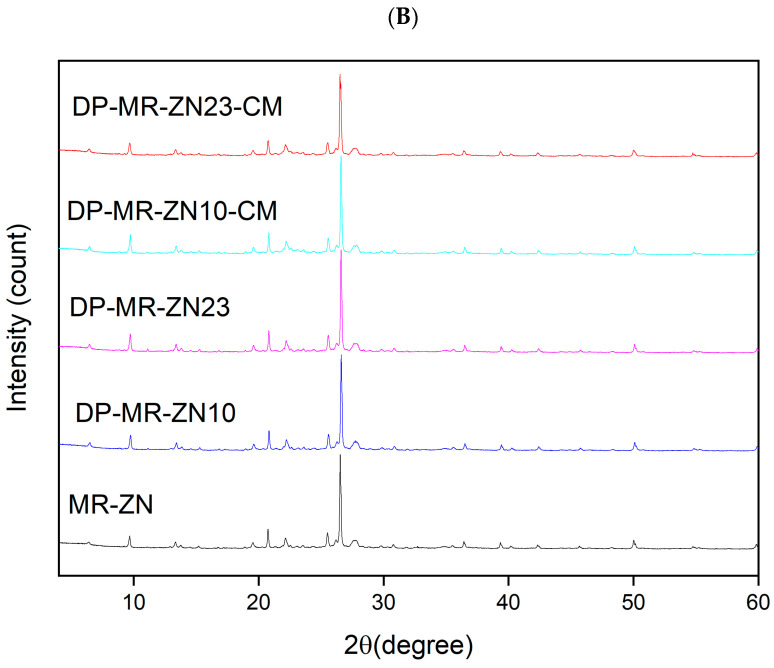
X-ray diffraction (XRD) patterns of (**A**) natural mordenite (MR) and zinc-modified mordenite (MR-ZN), (**B**) mordenite loaded with 10 mg donepezil (DP-MR-ZN10), mordenite loaded with 23 mg donepezil (DP-MR-ZN23), and donepezil-loaded mordenite formulated with microcrystalline cellulose (DP-MR-ZN10-CM and DP-MR-ZN23-CM); Mordenite (*), Sodium Clinoptilolite (+), Montmorillonite (^o^), and Heulandite (-).

The X-ray diffraction (XRD) patterns of natural mordenite (MR) and zinc-modified mordenite (MR-ZN), shown in Figure 5, were analyzed to assess crystallinity and structural integrity following zinc modification. The diffraction peaks of MR corresponded to characteristic reflections of mordenite (Ref. No. 9003354 and space group C m c 21), sodium clinoptilolite (Ref. No. 1532903 and space group C 1 2/m 1), and montmorillonite (Ref. No. 7240309 and space P 1 21/c 1), as indexed in the Crystallography Open Database [25]. The reflections observed at 2θ values of approximately 6.5°, 9.7°, 13.4°, 15.3°, 19.6°, 20.8°, 22.2°, 25.6°, 26.6°, 27.8°, 29.8°, 30.9°, 36.5°, 39°, 40.3°, 42.4°, 45.6°, 48.4°, 50.1°, 54.9° align with previously reported mordenite structures belonging to the orthorhombic crystal system (a[Å] = 18.3, b[Å] = 20.5 and c[Å] = 7.5). Reflections near 9.8°, 11.1°, 23.3° and 44.4° were consistent with sodium clinoptilolite, which adopts a monoclinic crystal system (a[Å] = 17.2, b[Å] = 18.1 and c[Å] = 7.4), while reflections near 9.3°, 18.0° and 33.5° matched reported montmorillonite patterns, also monoclinic (a[Å] = 5.7, b[Å] = 19.0 and c[Å] = 10.9). The results revealed that the sample consisted mainly of mordenite (50.0%) and sodium clinoptilolite (43.0%), accompanied by a small fraction of montmorillonite (6.1%) and other minor phases (0.9%). Although the predominance of zeolitic phases such as mordenite and clinoptilolite over clay minerals may appear unusual, similar high zeolite-to-clay ratios have been reported for volcanic tuffs from Ecuador and other Andean regions. These materials are typically rich in secondary mordenite and clinoptilolite formed through prolonged hydrothermal alteration and leaching processes that minimize quartz or feldspar content, resulting in zeolite-rich frameworks with limited amorphous or clay phases [26]. This composition confirms that mordenite is the dominant crystalline phase. Following ion exchange with Zn^2+^ incorporation, the XRD pattern of mordenite exhibited slight shifts or changes in peak intensities, reflecting Zn^2+^ incorporation. Zinc ions substituted Na^+^, Ca^2+^, or K^+^ within the mordenite framework, causing structural distortion due to their different charge and coordination preferences. Notably, a transition from sodium clinoptilolite to heulandite was observed post-modification. This transformation, induced by ion exchange between monovalent sodium (Na^+^) and divalent zinc (Zn^2+^), is attributed to differences in Si/Al ratios; clinoptilolite typically has Si/Al > 4.0, while heulandite has Si/Al < 4.0 [27]. The substitution of Na^+^ with Zn^2+^ lowers the Si/Al ratio by effectively increasing the influence of Al sites, favoring heulandite formation, which is more stable in the presence of divalent cations [28]. As confirmed by chemical composition analysis, the pristine zeolite had a Si/Al ratio of 4.5, while the modified sample had a ratio of 4.2. The higher charge density of Zn^2+^ may promote structural stabilization of heulandite-like arrangements under specific environmental conditions. Although clinoptilolite generally possesses higher thermal stability, Zn^2+^ incorporation can induce partial reorganization toward heulandite-like structure [29]. The XRD pattern of MR-ZN displayed characteristic reflections of heulandite (Ref. No. 1544852, and space group C 1 2 /m 1) with peaks near 2θ = 9.2°, 11.1°, 16.7°, 23.9°, 25.5° and 26.5°, consistent with monoclinic crystal system with unit cell dimensions of a[Å] = 17.6, b[Å] = 19.2 and c[Å] = 7.5. Additionally, the shift in the hkl (110) reflection at 2θ = 6.4° and an increase in d-spacing from 13.65 Å in natural mordenite to 14.55 Å in zinc-modified mordenite indicate structural expansion. This expansion, due to Zn^2+^ incorporation, enhances the zeolite’s adsorptive properties by improving pore accessibility and increasing active site density [30], which can be favorable for donepezil adsorption, as observed for other drugs in zeolitic matrices [14].

**Figure 6:**
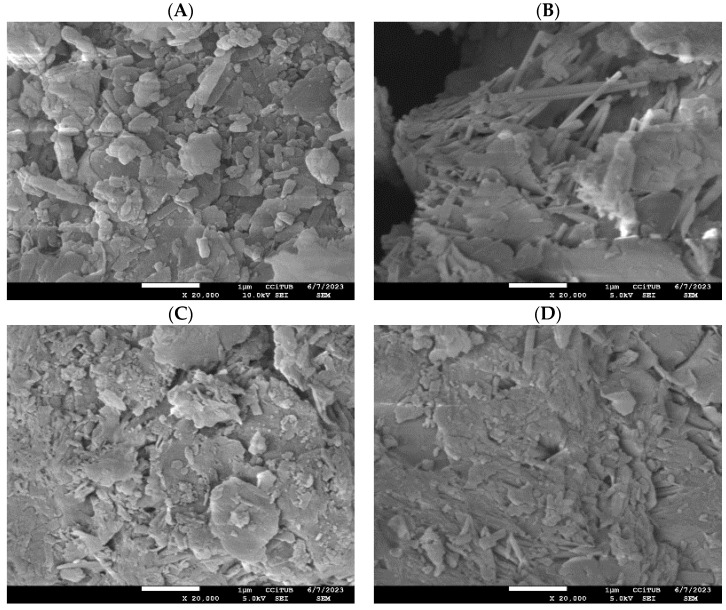

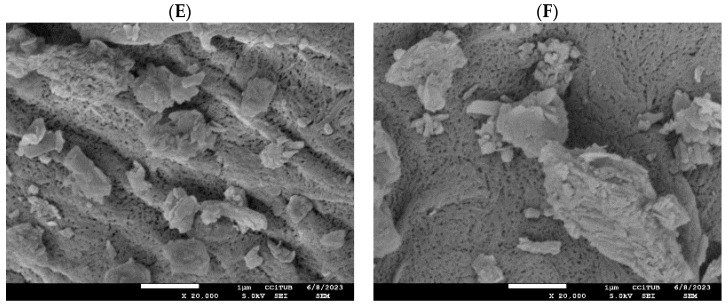
Morphological characterization: (**A**) natural mordenite, and (**B**) zinc-modified mordenite (MR-ZN), (**C**) mordenite loaded with 10 mg donepezil (DP-MR-ZN10), (**D**) mordenite loaded with 23 mg donepezil (DP-MR-ZN23), and (**E**,**F**) and donepezil-loaded mordenite formulated with microcrystalline cellulose (DP-MR-ZN10-CM and DP-MR-ZN23-CM). Scale bar = 1 µm. Magnification: ×8000.

The X-ray diffraction (XRD) patterns for zinc-modified mordenite (MR-ZN) samples were analyzed to assess the crystallinity and structural integrity of the zeolite following donepezil (DPZ) adsorption and the incorporation of microcrystalline cellulose (Figure 6). The XRD results confirm that zinc-modified mordenite retains its structural integrity after incorporation of donepezil and cellulose, as no new crystalline phases were observed. The characteristic reflections of mordenite remain prominent in DP-MR-ZN10 and DP-MR-ZN23, indicating that the zeolite framework remains largely intact. Minor variations, such as increased peak intensities around 2θ: 19.6°, 20.8°, and 22.2°, observed with increasing donepezil content, suggest successful drug adsorption, as previously reported in other donepezil-impregnated materials [6,31]. Given the relatively large size of donepezil (14–15 Å in length, 7–8 Å in width, and 5–6 Å is height), its accommodation within the zeolite micropores may cause slight lattice distortions without complete framework incorporation. These observations are consistent with the known channel dimensions of mordenite, which contains primary twelve-membered ring channels (6.7 Å × 7.0 Å), and secondary eight-membered ring channels (2.6 Å × 5.7 Å) [32]. In contrast, clinoptilolite features three types of channels: type A (ten-membered rings, 3 Å × 7.6 Å), type B (eight-membered rings, 3.3 Å × 4.6 Å), and type C (eight-membered rings, 2.6 Å × 4.7 Å), while heulandite exhibits channels formed by ten-membered rings (3.1 Å × 7.5 Å), and eight-membered rings (3.6 Å × 4.6 Å and 2.8 Å × 4.7 Å) [33]. The addition of cellulose, particularly in samples DP-MR-ZN10-CM and DP-MR-ZN23-CM, resulted in a reduction in peak intensity with increasing microcrystalline cellulose content. This effect, observed around peaks at 2θ values of 20.8°, 25.6°, and 26.6°, is attributed to the crystalline components of cellulose. Moreover, the amorphous region of cellulose generates a halo peak around 21° [34]. Given the large average particle diameter of microcrystalline cellulose (~50 Å) [35], it does not integrate into the mordenite crystalline lattice but likely interacts with the zeolite surface, perturbing the diffracted intensity while preserving the core structure of the zeolite.

Figure 6 presents the morphology of the samples examined in this study: natural mordenite (MR) and zinc-modified mordenite (MR-ZN). The scanning electron microscopy (SEM) images primarily reflect the crystalline framework of mordenite, which is characterized by flaky structures composed of fine-grained, irregularly shaped particles with a marked tendency to agglomerate [36]. In certain regions, the presence of clinoptilolite morphology is evident through plate-like crystal morphologies, indicating the coexistence of both mordenite and clinoptilolite phases. In the natural zeolite (MR), visible entries to pore channels and internal cavities, along with regions of loosely aggregated particles, further support the microporous nature of the mordenite framework [14]. In contrast, the zinc-modified mordenite (MR-ZN) presents a denser and more compact surface texture, with larger agglomerates and smoother crystallite edges. These changes are attributed to the incorporation of zinc ions at extra-framework positions, inducing slight structural rearrangements that reduce the surface roughness and occlude some pore openings. These changes indicate that Zn^2+^ exchange enhances particle cohesion while decreasing apparent porosity and altering the channel structure, consistent with the generation of additional Lewis acid sites within the framework, a behavior also reported in a copper-modified natural mordenite [37].

Figure 6C–F presents the morphology of donepezil-loaded mordenite with 10 mg (DP-MR-ZN10) and 23 mg (DP-MR-ZN23), donepezil formulated with microcrystalline cellulose (DP-MR-ZN10-CM), and donepezil formulated with microcrystalline cellulose (DP-MR-ZN23-CM). In both DP-MR-ZN10 and DP-MR-ZN23, donepezil molecules appear mainly adsorbed onto the external surface of the zeolite particles, resulting in smoother regions and less sharply defined crystallite edges, while the overall zeolitic framework remains intact. These effects are consistent with surface adsorption dominating over deep diffusion into the micropores [38]. In contrast, the formulations containing microcrystalline cellulose (DP-MR-ZN10-CM and DP-MR-ZN23-CM) display more pronounced morphological changes. The SEM images show a continuous coating over the zeolite particles, which imparts a smoother texture and visibly reduces surface porosity. This coating effect is characteristic of microcrystalline cellulose, whose amorphous–crystalline domains tend to mask the underlying crystalline features of the zeolite; notably, while the cellulose layer modifies the surface accessibility, it does not disrupt the primary mordenite framework, confirming that the zeolite preserves its structural integrity in the hybrid formulations.

The FTIR spectra of natural mordenite (MR) and zinc-modified mordenite (MR-ZN) are presented in Figure 6. The natural zeolite (MR) displays characteristic vibrational bands corresponding to Si-OH silanol groups at 3750 cm^−1^ and bridging hydroxyl groups (Si-OH-Al) at approximately 3619 cm^−1^. Broad absorption bands between 3500–3000 cm^−1^ and a peak near 1640–1620 cm^−1^ are attributed to physically adsorbed water, commonly observed in zeolitic materials [39]. Additional characteristic bands include a prominent peak at 1007 cm^−1^ (Si-O-Si asymmetric stretching), 694–777 cm^−1^ (Si-O symmetric stretching), and a band near 450 cm^−1^ (Si-O bending, not shown here). These features are typical of mordenite and confirm the preservation of the tetrahedral SiO_4_ and AlO_4_ units within the zeolite framework. Cation exchange of zeolites generally does not lead to significant shifts in FTIR band positions [40]. Consistently, the zinc-modified mordenite (MR-ZN) retains the characteristic vibrational bands, confirming the structural stability of the zeolite after modification. This behavior is consistent with previous reports for other transition metals introduced into mordenite, where ion exchange modifies the local environment around silicon and aluminum atoms, inducing only subtle framework changes [14,37]. However, a slight reduction is observed in the OH band within the 3500–3000 cm^−1^ region, which is attributed to hydroxyl groups associated with metal cations. Moreover, since the modification was carried out at intermediate pH (≈5–7), hydrolyzed species such as ZnOH^+^ and Zn_2_OH^3+^ may coexist with acetate complexes (Supplementary Material, Figure S1). This suggests that, in addition to ion exchange, Zn^2+^ may coordinate with bridging oxygens (Si–O–Al) of the zeolite lattice. Such coordination aligns with the experimentally observed increase in Lewis acidity, evidenced by the shift in the point of zero charge (pHPZC) from 8.7 for MR to 7.0 for MR–ZN, as previously reported in studies where a decrease in pHPZC is associated with the formation of additional Lewis acid sites [41], that enhance interactions with functional groups of adsorbed molecules. A shift of the Si-O-Si asymmetric stretching band toward lower wavenumbers is also observed, indicative of framework distortions caused by Zn^2+^ incorporation. This phenomenon can be explained by changes in electron density and bond strength within Si–O–T (T = Si or Al) linkages. Furthermore, variations in the intensity and position of bands in the 600 to 800 cm^−1^ region are associated with Zn-O stretching vibrations, confirming the successful incorporation of Zn^2+^ into the zeolite framework [42]. These features reflect the presence of exchangeable cations at specific sites within the zeolite [39], and extra-framework Zn^2+^ enhancing the Lewis acidity [43], promoting stronger interactions between the framework and donepezil.

**Figure 7:**
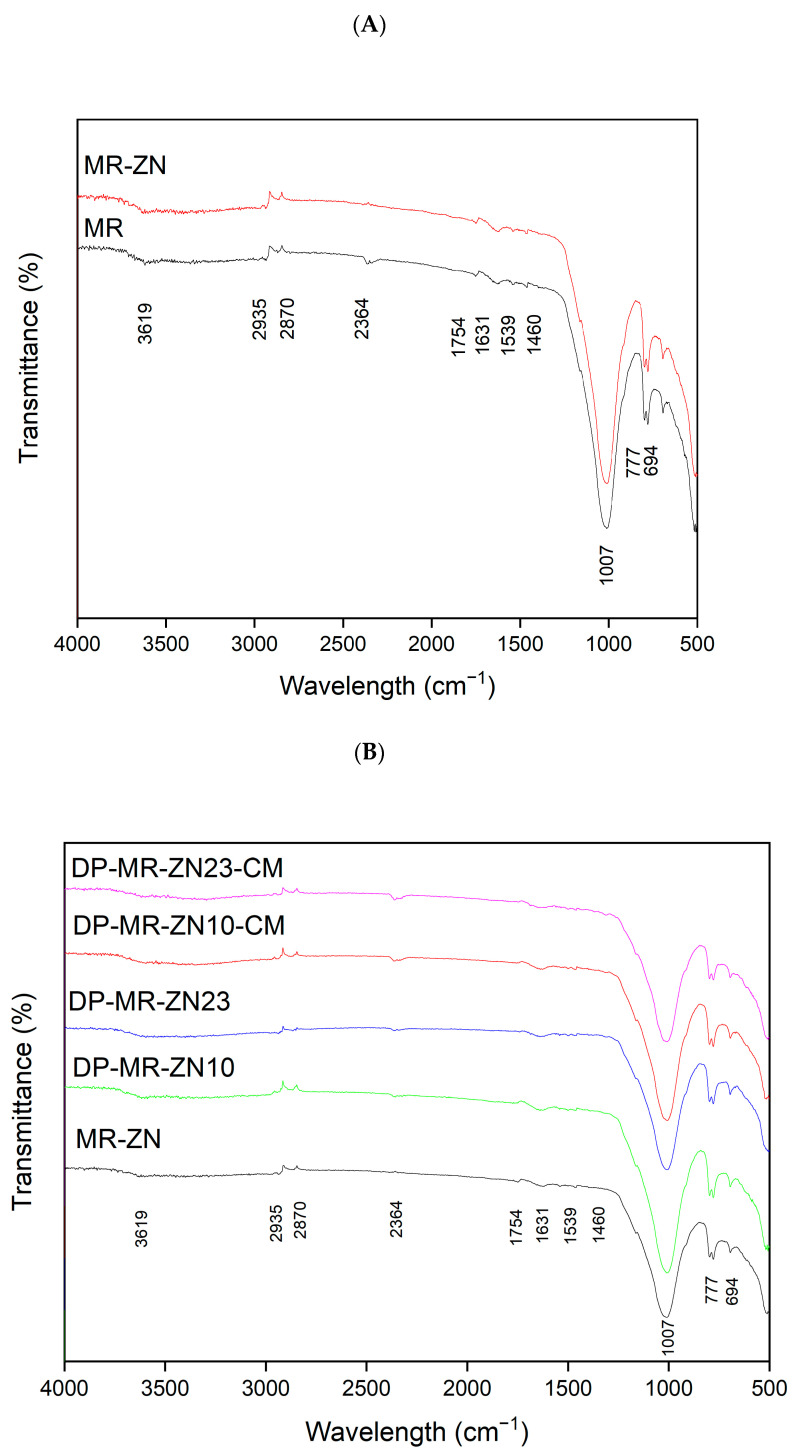
FTIR spectra of (**A**) natural mordenite (MR) and mordenite modified with zinc (MR-ZN), (**B**) mordenite loaded with 10 mg donepezil (DP-MR-ZN10), mordenite loaded with 23 mg donepezil (DP-MR-ZN23), and donepezil-loaded mordenite formulated with microcrystalline cellulose (DP-MR-ZN10-CM and DP-MR-ZN23-CM).

FTIR analysis of the zinc-modified mordenite revealed notable spectral changes following the incorporation of donepezil and microcrystalline cellulose (Figure 7). The FTIR spectrum of donepezil features sharp absorption bands at 1685 cm^−1^, 1498 cm^−1^ and 1308 cm^−1^, corresponding to C=O stretching, C-N-C vibration, and C-H vibrations, respectively [31,44]. The spectra of donepezil-loaded zeolite samples (DP-MR-ZN10 and DP-MR-ZN23) show changes in absorption bands at 1539, and 1631 cm^−1^, associated with the drug’s functional groups (C=O, and C-N-C), and attenuation of the mordenite band at 1007 cm^−1^ [45]. The observed decrease in the asymmetric Si–O–T (T = Si or Al) stretching vibrations upon donepezil incorporation suggests strong interactions between the drug and the zeolite framework, likely involving coordination of the drug’s amine or carbonyl groups with Zn^2+^ sites. This coordination can cause localized electronic perturbations and reduced vibrational activity [46]. Additional modifications were observed in the O–H stretching region (3550 and 3100 cm^−1^), associated with hydrogen bonding between the carbonyl group of donepezil and silanols or coordinated water molecules. The C-H stretching range (2840 and 3000 cm^−1^) reflects dispersive and van der Waals interactions without covalent bond cleavage [44]. The addition of microcrystalline cellulose (DP-MR-ZN10-CM and DP-MR-ZN23-CM) resulted in slight changes in the FTIR spectra. However, no significant shifts in the Si–O–Si bands were observed, indicating that cellulose is physically adsorbed on the surface without chemically modifying the zeolite framework. Decreased in intensity at 2935 cm^−1^ and 1007 cm^−1^ were attributed to C–H and C–O–C stretching, with increasing drug and microcrystalline cellulose content; variations at 1631 cm^−1^, 1460 cm^−1^, and 1374 cm^−1^ (assigned to C=O stretching, CH_2_ bending, and C–H asymmetric stretching, respectively) [47] support this interpretation. These results agree with previous studies on drug-zeolite interactions [31], indicating that donepezil interacts with both external and internal sites of the zeolite through physisorption and chemisorption by hydrogen bonding and coordination mechanisms of donepezil to Zn^2+^. Thus, Zn^2+^-mediated chemisorption accounts for stronger and more specific interactions with the zeolitic framework.

### 2.3. Functional Evaluation of DPZ Adsorption on Zinc-Modified Mordenite: Loading, Release, Kinetics, and Mechanistic Insights

#### 2.3.1. Drug Loading of Donepezil onto Zinc-Modified Mordenite Formulations

**Figure 8:**
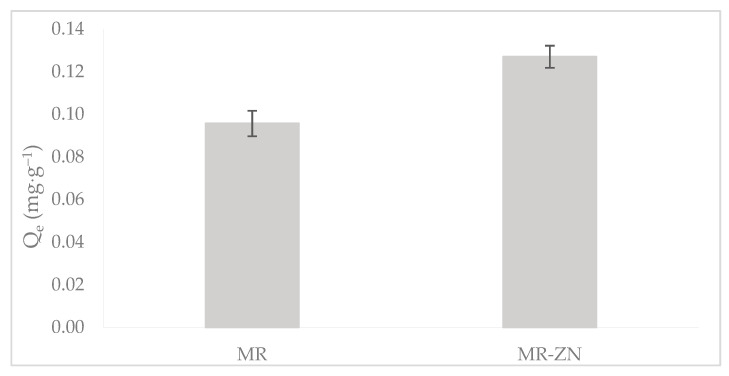
Effect of zinc modification on the adsorption of donepezil: comparison between natural mordenite and MR–ZN at pH 8.

Zinc incorporation effectively influenced the adsorption of donepezil, which agrees with the above-discussed characterization data (Figure 8).

The observed increase in donepezil uptake for MR–ZN in comparison to natural mordenite (MR) can be directly correlated with the structural and surface modifications induced by Zn^2+^. As previously discussed, the reduction of the point of zero charge pH_PZC_ (from 8.6 to 7.0) reflects the generation of new Lewis acidic sites and an improvement in surface characteristics (from 62.1 for MR to 85.4 m^2^·g^−1^ for MR-ZN), both of which favor stronger interactions with the functional groups of donepezil. These findings corroborate the spectroscopic evidence of Zn–framework interactions (FTIR) and the creation of additional coordination sites, thereby validating that zinc modification not only alters the acid–base properties of the zeolite but also enhances its adsorption performance.

The results obtained from the drug loading efficiency studies demonstrated a high capacity of zinc-modified mordenite (MR-ZN) to adsorb donepezil. Specifically, the DP-MR-ZN10 sample exhibited a drug loading efficiency of 95 ± 6.2%, while DP-MR-ZN23 achieved 94 ± 3.8%. The high drug loading efficiency observed in this study for donepezil can be attributed to the favorable alignment between the physicochemical properties of the drug and the structural characteristics of the zinc-modified mordenite. Particularly, the suitable molecular size of donepezil (415.96 g/mol) allows partial penetration into the larger pores and channels of mordenite, while also favoring adsorption on the external surface. Moreover, its moderate polarity and cationic nature, with the presence of a basic amine group, promote strong interactions with the acidic sites of the zeolite. Additionally, its solubility in the hydroalcoholic medium further facilitates its diffusion into the porous network during the adsorption phase [48,49].

#### 2.3.2. Controlled Release Characteristics of Donepezil in Simulated Gastrointestinal Conditions

**Figure 9:**
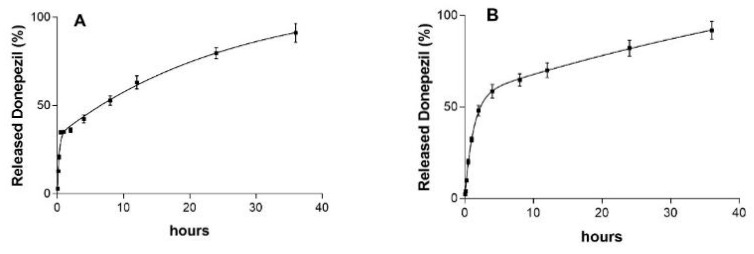
Release kinetics of donepezil from the zeolite-based delivery system. (**A**) Capsules of mordenite loaded with 10 mg donepezil (DP-MR-ZN10-CM), (**B**) Capsules of mordenite loaded with 23 mg donepezil (DP-MR-ZN23-CM).

Quality control in drug delivery focuses on ensuring that pharmaceutical products release their active ingredients at the intended rate while also exploring new release models, both of which are crucial for ensuring drug efficacy and safety [50]. In both cases (DP-MR-ZN10-CM and DP-MR-ZN23-CM capsules), a rapid drug release of approximately 36% and 48% of donepezil was observed during the first two hours, respectively (Figure 9). This initial burst release can be attributed to the desorption of drug molecules weakly bound to the external surface of the zeolite by physical adsorption. However, it could also be due to the acidic conditions under which the release occurred (pH 1.2). In contrast, at intestinal pH, drug release slowed; however, it remained sustained, with 80% and 82% of DP-MR-ZN10-CM and DP-MR-ZN23-CM being released after 24 h of testing, respectively. The release of both formulations was fitted to a second-order kinetic model. This type of kinetic behavior is commonly observed in systems where drug release is influenced by the degradation or erosion of the delivery matrix, or by interactions between the drug and the components of the formulation, in this case, mordenite, microcrystalline cellulose, and donepezil [51]. Moreover, the release rate can be complex and may vary throughout the process, potentially accelerating or decelerating at different stages depending on factors such as the drug concentration and environmental conditions within the delivery system, including pH in this case [52].

Furthermore, this mordenite-based delivery system provides an alternative to conventional polymeric excipients, offering advantages such as protecting the drug from degradation [12,53], which could improve its stability both in storage and in the gastrointestinal tract. Therefore, the zinc-modified mordenite system demonstrates significant potential as a controlled-release vehicle for drugs, supporting its further development as an alternative drug delivery strategy for improving treatment adherence and therapeutic outcomes in AD [14,54].

According to the United States Pharmacopeia (USP) specifications for oral solid dosage forms, at least 80% of the labeled drug content should be released to ensure therapeutic efficacy. The release profiles obtained in this study, achieving 80% and 82% release for the 10 mg and 23 mg formulations, respectively, therefore meet this pharmacopoeial requirement and confirm that the developed zeolite-based delivery system provides a therapeutically relevant dosage under simulated gastrointestinal conditions.

#### 2.3.3. Evaluation of Kinetic Models for Donepezil Adsorption on Zinc-Modified Mordenite

**Figure 10:**
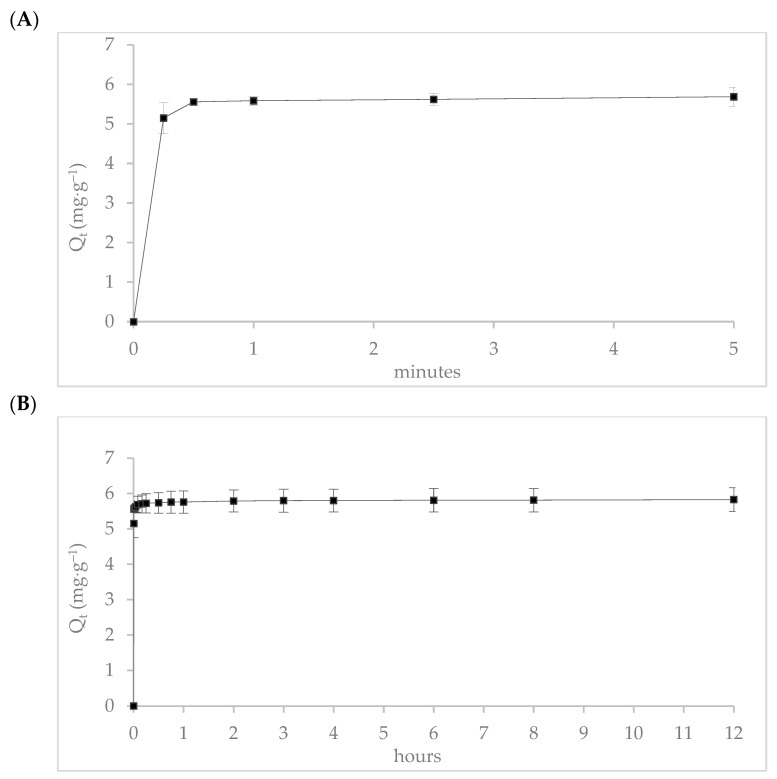
Adsorption kinetics of donepezil onto zinc-modified mordenite (MR-ZN): (**A**) rapid adsorption within the first 5 min indicating surface saturation dynamics, and (**B**) extended adsorption behavior over 12 h confirming equilibrium state and adsorption stability.

The adsorption kinetics of donepezil onto zinc-modified mordenite were evaluated over two specific time intervals. During the initial 5 min (Figure 10A), 95% of donepezil was adsorbed within the first 15 s, achieving an adsorption capacity of 5.2 mg∙g^−1^. Adsorption gradually increased to 98% over an 8 h period, reaching a capacity of 5.8 mg∙g^−1^ (Figure 10B), indicating that equilibrium was essentially attained, with negligible variation in subsequent measurements. This rapid kinetic profile suggests a strong affinity of donepezil for the mordenite surface.

Experimental data were fitted to the kinetic models summarized in Table 4. The pseudo-first-order model showed poor correlation (R^2^ = 0.59) and significantly underestimated the equilibrium adsorption capacity (Q_e_ = 0.11 mg∙g^−1^), suggesting that physisorption is not the dominant mechanism. Although the pseudo-first-order (PFO) model is typically associated with physisorption processes involving reversible adsorption through weak van der Waals and hydrogen bonding interactions [55], the low correlation obtained in this study confirms that such physical interactions play only a minor role in the overall adsorption mechanism of donepezil onto MR–ZN. Conversely, the pseudo-second-order model best described the experimental data, with an R^2^ of 0.99 and a theoretical adsorption capacity (Q_e_ = 5.58 mg∙g^−1^) closely matching the experimental value. The excellent fit of the experimental data to the pseudo-second-order kinetic model suggests that the adsorption of donepezil on MR–Zn is primarily governed by chemisorption processes. This model assumes that the rate-limiting step involves surface interactions between adsorbate and adsorbent through electron-sharing or electron-exchange mechanisms [55]. Such behavior has been widely associated with chemisorptive interactions in metal-modified adsorbents, supporting the strong and specific binding of donepezil onto the zinc-modified mordenite surface. The intraparticle diffusion model revealed four distinct linear regions characterized by decreasing diffusion rate constants (K_t1_ to K_t4_), suggesting a multi-stage adorption process. The first stage (K_t1_ = 64.61 mg∙g^−1^∙h^−1/2^, R^2^ = 0.92) corresponds to rapid transport of donepezil molecules from the bulk solution to the external surface of the zeolite, typically governed by boundary layer effects. This high initial diffusion rate confirms strong affinity and rapid uptake of donepezil. Subsequent regions (K_t2_ = 0.03, K_t3_ = 0.05, and K_t4_ = 0.01 mg∙g^−1^∙h^−1^/^2^, R^2^ between 0.90 and 0.99) represent progressively slower diffusion into the internal pores of the zeolite. These phases likely involve transport through the complex pore network, where electrostatic interactions become increasingly relevant. The molecular dimensions of donepezil (approximately 14–15 Å in length) are comparable to the pore size of mordenite (~6.5–7.0 Å in diameter), suggesting that complete internal diffusion may be partially restricted or occur primarily in expanded channels or structural defects. This multistep behavior is typical of microporous materials, where rapid external adsorption is followed by slow internal diffusion. The decreasing diffusion rates indicate gradual saturation of pore space and a diminishing diffusion-driving force as equilibrium is approached. The film diffusion model, which assesses resistance across the external boundary layer, yielded a diffusion coefficient (D_F_ = 4.3 × 10^−9^ m^2^∙h^−1^) and a low correlation coefficient (R^2^ = 0.66), indicating that film diffusion plays a minor role. This aligns with the observed rapid adsorption (within 1 min), suggesting minimal external mass transfer resistance and effective interaction between donepezil molecules and the adsorbent surface, confirming that the boundary layer is not a limiting factor. The particle diffusion model, describing diffusion within the solid matrix, provided a diffusion coefficient (D_P_ = 2.9 × 10^−11^ m^2^∙h^−1^) with a moderate R^2^ value of 0.72. This indicates that while internal diffusion contributes to overall adsorption process, it is not the primary rate-limiting step. However, the model supports the occurrence of intraparticle transport, particularly in zeolite structures with mesopores or structural defects allowing penetration of larger molecules such as donepezil. Additionally, the partial substitution of Na^+^ by Zn^2+^ ions may enhance the acidity and polarity of internal adsorption sites, strengthening interactions with the amine and carbonyl groups of donepezil. This internal chemisorption contributes to the gradual uptake observed in the later stages of the intraparticle diffusion curve.

The observed rapid adsorption is advantageous for drug-loading applications, enabling efficient encapsulation within short contact times. Furthermore, the high affinity and stable binding suggest that zinc-modified mordenite is a promising can matrix for the controlled release of donepezil in therapeutic systems.

#### 2.3.4. Mechanistic Interpretation of Donepezil Adsorption on Zinc-Modified Mordenite

**Figure 11:**
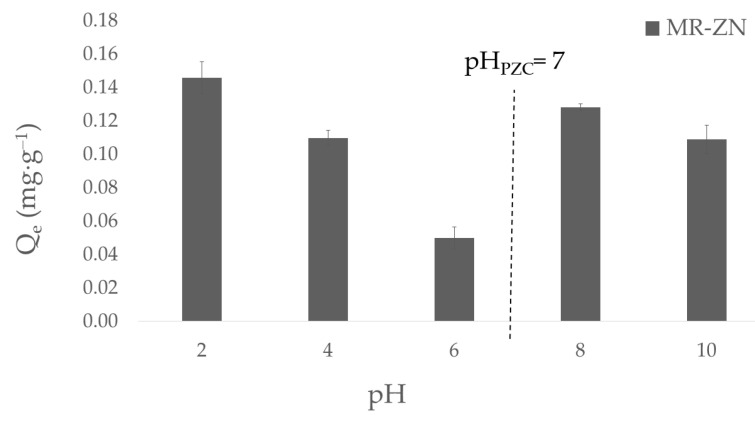
Effect of pH on the adsorption capacity of donepezil onto zinc-modified mordenite (MR–ZN).

The adsorption of donepezil on natural mordenite and zinc-modified mordenite (MR–ZN) as a function of pH is shown in Figure 11.

A clear dependence of drug uptake on pH is observed, which can be rationalized by considering both the surface charge of the zeolite and the ionization state of donepezil. The point of zero charge (pH_PZC_) of MR–ZN is 7.0, implying that the zeolite surface is positively charged at pH values below this threshold and negatively charged at higher pH. The speciation profile of donepezil (DPZ) as a function of pH reveals a progressive shift in the distribution of ionic species, which directly influences its adsorption behavior on the zeolitic surface. At strongly acidic conditions (pH < 2–3), donepezil exists predominantly in its dicationic (DPZ^2+^) and monocationic (DPZ^+^) forms due to full protonation of the tertiary amine and secondary basic sites, resulting in a highly charged and soluble species. As the pH increases to the range of 3–6, protonation decreases and a neutral or zwitterionic form gradually emerges, while the fraction of the dicationic species diminishes. Around physiological pH (≈7.4), the distribution becomes more complex, with approximately 45% of the molecules in the neutral form, ~35% as monocationic (DPZ^+^), and ~20% as anionic (DPZ^−^) species, indicating the coexistence of cationic, neutral, and anionic populations in equilibrium. In the alkaline region (pH 8–10), the fraction of cationic species declines sharply as the tertiary amine becomes deprotonated, leading to an increasing prevalence of the neutral form, followed by the anionic species at higher pH values. Beyond pH 10–11, the molecule is almost entirely in its anionic (DPZ^−^) form. At acidic condition, adsorption was relatively high due to the prevalence of protonated donepezil species (DPZ^2+^/DPZ^+^), whose solubility and ability to form hydrogen bonds and van der Waals interactions favored stabilization within the zeolitic framework. However, as the pH approached neutrality (pH 4–6), the adsorption capacity decreased, reflecting electrostatic repulsion between the positively charged zeolite surface (pH < PZC) and the cationic amine of DPZ. Most importantly, at alkaline conditions (pH 8–10), adsorption significantly increased, reaching its maximum at pH 10. The experiments performed at pH 8 confirm this trend and highlight the mechanistic relevance of this environment. At this pH, the MR–ZN surface is negatively charged (pH > PZC), while donepezil remains partially protonated (~30–40%), thus enabling strong electrostatic attraction. Simultaneously, the proximity to the basic pKa of DPZ (7.97) allows partial deprotonation of the tertiary amine, exposing its lone electron pair for coordination with Zn^2+^ centers. This coordination, together with interactions involving the carbonyl oxygen, provides direct evidence of chemisorption, reinforcing the role of both electrostatic stabilization and Lewis acid–base interactions as the dominant mechanisms under the selected experimental conditions. Therefore, the adsorption profile as a function of pH reflects the contribution of physisorption and chemisorption, supporting the kinetic modeling results where the pseudo-second-order model exhibited an excellent fit (R^2^ > 0.99), consistent with a chemisorption-controlled process, while the pseudo-first-order model showed poor correlation (R^2^ = 0.59), confirming that purely physical adsorption mechanisms are not predominant.

The adsorption of donepezil onto zinc-modified mordenite (MR–ZN) thus proceeds through a combined mechanism involving both chemisorption and physisorption. Chemisorption predominates due to the coordination of the drug’s amine and carbonyl groups with Zn^2+^ Lewis acid sites, as evidenced by FTIR spectral shifts and the excellent fit of the kinetic data to the pseudo-second-order model (R^2^ > 0.99). In parallel, weaker physical interactions such as hydrogen bonding and van der Waals forces contribute to molecular stabilization within the zeolitic channels. This combined mechanism reconciles the experimental and theoretical findings, indicating that while chemisorption governs the primary binding process, physisorption plays a secondary but complementary role enabling reversible and sustained drug release.

##### Physical Interactions of Donepezil

At the experimental conditions pH 8, and in accordance with the speciation diagram of donepezil (Supplementary Material, Figure S2), the tertiary amine group of donepezil is protonated (–N^+^(CH_3_)_2_), enabling strong coulombic attractions with negatively charged oxygens from the Si–O–Al framework and with exchangeable Zn^2+^ cations within the mordenite structure. The electrostatic interactions are reversible and characteristic of physical adsorption processes, as similar stabilization of protonated amines in mordenite has been reported by computational studies [56,57,58]. Moreover, the carbonyl group (C=O) of donepezil can engage in hydrogen bonding with surface silanol groups of the zeolite, while water molecules coordinated to Zn^2+^ may act as donors or acceptors. These hydrogen bonds are relatively weak and do not require covalent bond formation or cleavage, thus falling under physisorption. Computational studies have demonstrated the prevalence of hydrogen-bonding networks involving silanol nests in zeolites [59,60]. The aromatic rings and aliphatic chains of donepezil interact with the hydrophobic regions of mordenite channels through dispersion forces and confinement effects. These non-specific Van der Waals interactions facilitate molecular retention inside the pores without the need for strong chemical bonding, representing a purely physical mechanism. Previous adsorption studies of aromatic hydrocarbons on MFI and Y zeolites have shown that hydrophobic confinement and dispersive interactions are the dominant driving forces for uptake in microporous frameworks [61,62].

##### Chemical Coordination of Donepezil

The presence of extra-framework Zn^2+^ in modified mordenite generates strong Lewis acid sites capable of coordinating directly with electron-donating groups of donepezil. The tertiary amine nitrogen can act as a Lewis base, forming stable N → Zn^2+^ coordination bonds, while the carbonyl oxygen can also interact with Zn^2+^ to yield Zn–O=C complexes. These interactions are stronger and more specific than hydrogen bonding or electrostatic forces, corresponding to true chemisorption. Evidence for such coordination has been extensively reported in Zn-exchanged zeolites, where pyridine adsorption studies confirm the generation of Lewis acidity through FTIR bands [63]. Similar findings in Zn-BEA and Zn-ZSM-5, as determined by FTIR and TPD measurements, demonstrate the role of Zn^2+^ in establishing coordination with nitrogen- and oxygen-containing molecules. Computational and spectroscopic investigations further support that Zn^2+^ behaves as a Lewis acid center capable of forming strong coordinative complexes with probe molecules [63,64]. Beyond creating discrete Lewis acid sites, Zn^2+^ alters the acid-site distribution and local polarization of framework oxygens, collectively reinforcing donor–acceptor interactions. These findings highlight the synergistic role of Zn^2+^ in modifying the acid site distribution and reinforcing coordinative interactions, which are critical for stable donepezil adsorption [65,66,67].

### 2.4. Implications of Zinc-Modified Mordenite as Drug Delivery System: Advantages and Disadvantages

The incorporation of donepezil into zinc-modified mordenite presents a promising strategy for drug delivery, particularly in terms of structural stability, surface interaction potential, and sustained release behavior. Zeolites such as mordenite are characterized by their high thermal and chemical stability, microporous frameworks, and ion-exchange capacity, which make them suitable candidates for hosting pharmaceutical agents [7,68]. In the present study, DFT simulations and physicochemical characterizations confirmed that donepezil interacts with the MR-ZN framework predominantly through hydrogen bonding and Van der Waals forces, suggesting physical adsorption without compromising the structural integrity of the host. This is advantageous for maintaining drug stability and avoiding premature degradation during storage or transit through physiological environments.

Compared to traditional delivery systems such as mesoporous silica, which have been widely used for the treatment of Alzheimer’s disease due to their high surface area and tunable pore size [69], mordenite exhibits superior framework stability and potential biocompatibility. For instance, mesoporous silica nanoparticles exhibit a wide-ranging surface area due to their porous structure, providing abundant space for drug encapsulation and functional molecule binding, along with a notable ability to efficiently traverse the blood–brain barrier (BBB) [69].

The zinc-modified mordenite (MR–ZN) functions as an oral controlled-release carrier designed to protect donepezil during gastrointestinal transit and enable sustained, pH-responsive release under intestinal conditions. This mechanism helps maintain stable plasma concentrations, reduces dosing frequency, and minimizes toxicity peaks typically associated with conventional administration [5]. The presence of Zn^2+^ sites enhances drug–carrier affinity through coordination and electrostatic interactions, promoting gradual desorption and ensuring consistent therapeutic delivery. Acting as an inert and non-absorbable matrix, the zinc-modified zeolite facilitates prolonged pharmacological efficacy, improved bioavailability, and greater treatment adherence while being safely eliminated through the gastrointestinal tract after drug release [5].

However, the limitations of mordenite-based drug delivery systems must also be acknowledged. Due to the relatively small pore aperture of mordenite (≈6.5–7 Å), large molecules like donepezil (≈14–15 Å) experience steric hindrance, resulting in partial surface adsorption and limited diffusion into the micropores. This could reduce the loading capacity compared to other carriers such as metal–organic frameworks (MOFs) or polymeric nanoparticles, which possess larger pore volumes and flexible architectures [5]. For instance, ZIF-8-based systems have been reported to allow donepezil encapsulation with pH-responsive release profiles, offering enhanced bioavailability and controlled release triggered by gastrointestinal conditions [70].

Another disadvantage relates to the non-biodegradable nature of mordenite, which may limit its long-term biomedical applications unless appropriately functionalized or used in ex vivo settings. In contrast, biocompatible polymeric matrices such as PLGA or chitosan offer enzymatically degradable environments, which may be more suitable for systemic drug delivery applications [71,72]. Despite these limitations, the zinc-modified mordenite system presents several advantages, including enhanced Lewis acidity due to Zn^2+^ exchange, strong electrostatic interactions, and high resistance to structural collapse under physiological conditions. These characteristics position zinc-modified mordenite as a potential candidate for oral formulations requiring gradual and site-specific release of donepezil. Furthermore, the moderate adsorption energy (−26.4 kJ/mol) suggests a favorable balance between drug stability and desorption potential, essential for achieving sustained therapeutic levels without rapid clearance.

## 3. Materials and Methods

### 3.1. Materials

Donepezil was acquired from Capot Chemical (Hangzhou, China). Natural mordenite zeolite was provided by ZEONATEC Cia. Ltd.a (Guayaquil, Ecuador). Microcrystalline cellulose was obtained from Fagron Ibérica (Barcelona, Spain). Chemicals used for analytical experiments and dissolution tests were purchased from Fisher Scientific (Hampton, NH, USA). The water used in all experiments was purified using a Millipore Milli-Q purification system (Millipore Corporation, Burlington, MA, USA).

### 3.2. Pretreatment and Modification of Zeolite

The natural mordenite employed in this study underwent no chemical purification prior to modification, except for a washing step with deionized water to remove surface impurities. Initially, the zeolite was subjected to particle size reduction using a vibratory disc mill, followed by sieving with a #200 mesh to ensure a uniform particle size distribution. The resulting material was then modified by impregnation with a 1 M ZnCl_2_ solution at pH 7 for 24 h, maintaining a solid-to-liquid ratio of 1:5. This process was conducted under reflux conditions. After the impregnation step, the zinc-modified zeolite (MR-ZN) was thoroughly washed with distilled water until no residual chloride ions were detected. Following the zeolite modification with zinc, residual concentrations of Na, K, and Ca in the resulting solution were quantified by atomic absorption spectrometry to assess the extent of ion exchange. The modified material was subsequently dried and stored for further characterization and experimental use.

### 3.3. High-Performance Liquid Chromatography (HPLC)

Donepezil quantification was performed using a Waters HPLC equipped with 2487 (UV/Vis) Detector and a 717 Plus Autosampler (Waters, Milford, MA, USA). Chromatographic separation was conducted on a Kromasil C18 column (250 × 4.6 mm × 5 µm). The mobile phase consisted of a methanol:buffer mixture (50:50, *v*/*v*), filtered through a 0.45 µm polyvinylidene fluoride (PVDF) membrane (Millipore Corp., Madrid, Spain). The buffer was composed of 0.05 M potassium dihydrogen orthophosphate 0.05 M, water, and trimethylamine, with the pH adjusted to 2.5 ± 0.05 using orthophosphoric acid. The mobile phase was delivered at flow rate of 1.2 mL/min. An injection volume of 20 µL was used, and the analyte was detected at 268 nm. Data acquisition and analysis were performed using Empower 3 software—Build 3471 (Waters, Milford, MA, USA, 2010).

### 3.4. Donepezil Incorporation into Zeolite

#### 3.4.1. Influence of pH and Zinc Incorporation on the Adsorption Behavior of Donepezil onto Mordenite

Adsorption studies were carried out to evaluate the uptake of donepezil (DPZ) onto natural mordenite (MR) and zinc-modified mordenite (MR–ZN) under controlled conditions. The effect of pH on donepezil adsorption was investigated using MR–ZN to identify the optimal conditions for drug–zeolite interaction. For these assays, 350 mg of MR–ZN were added to 10 mL of a methanol–water solution (36:64 *v*/*v*) containing 5 mg·L^−1^ of donepezil. Donepezil exhibited complete solubility in a methanol–water mixture (36:64, *v*/*v*); this optimized solvent composition ensured full dissolution and facilitated effective adsorption onto the zeolite surface. The initial pH was adjusted to 2, 4, 6, 8, and 10 using 0.1 M HCl or 0.1 M NaOH. After 24 h of agitation, the suspensions were centrifuged, and the residual concentration of donepezil in the supernatant was quantified by HPLC, as described in Section 3.3. The adsorption capacity (q_e_, mg·g^−1^) was calculated from the difference between the initial and equilibrium concentrations. Based on these results, pH 8 was identified as the most favorable condition for donepezil adsorption. Subsequently, comparative adsorption tests were performed under identical conditions using natural and zinc-modified mordenite to assess the influence of zinc incorporation on the adsorption performance.

#### 3.4.2. Quantification of Donepezil Loading Efficiency onto Zinc-Modified Zeolite

To quantify the loading efficiency, 10 mg samples of donepezil-loaded zeolite containing 10 mg (DP-MR-ZN10) and 23 mg (DP-MR-ZN23) of donepezil were dispersed in 10 mL of methanol–water solution at pH 8. The amounts of zinc-modified mordenite (620 mg and 607 mg) were selected to obtain a total capsule weight of 700 mg, including 10 mg or 23 mg of donepezil and 70 mg of microcrystalline cellulose, ensuring full drug incorporation and uniform formulation properties. The suspensions were sonicated in an ultrasonic bath for 30 min. The concentration of donepezil extracted from the zeolite matrix was then determined by HPLC.

#### 3.4.3. Kinetic of Donepezil Adsorption onto Zinc-Modified Zeolite

Adsorption kinetics were assessed by equilibrating 350 mg of zinc-modified zeolite with 10 mL of methanol–water solution (36:64) containing 250 mg·L^−1^ of donepezil at pH 8. Aliquots were collected at predefined time intervals ranging from 15 s to 24 h. The residual concentration of donepezil was quantified by HPLC and recorded at each time point. The adsorption capacity (Q_t_, in mg of donepezil per gram of zeolite) at each interval was calculated using Equation (1).

(1) $$Q_{t}=\left(c_{i}-c_{f}\right)\times \frac{v}{m}$$

where c_i_ and c_f_ are the initial and final concentrations (mg·L^−1^), respectively; v is the volume of solution (L) and m is the mass of zeolite (g).

### 3.5. Donepezil Loading and Formulation Procedure

Two drug solutions equivalent to 10 and 23 mg of donepezil were prepared by dissolving the active compound in a methanol–water mixture (36:64 *v*/*v*) at pH 8. Corresponding amounts of 620 and 607 mg of zinc-modified zeolite were added to these solutions and equilibrated for 24 h. The suspensions were filtered to separate the solid and liquid phases. The remaining donepezil in the filtrate was quantified by HPLC. The drug-loaded zeolite (DP-MR-ZN10) and (DP-MR-ZN23) was dried at 35 °C and subsequently blended with 70 mg of microcrystalline cellulose. The final mixture were encapsulated into gelatin capsules for further testing.

### 3.6. Simulated Gastrointestinal Dissolution of Donepezil-Loaded Zeolite Formulations

A dissolution test was performed for both the 10 mg (DP-MR-ZN10) and 23 mg (DP-MR-ZN23) donepezil formulations using the Pharmatest PTWS 120D Dissolver. A sequential pH protocol was employed to simulate gastrointestinal transit conditions. Initially, capsules were immersed in a hydrochloric acid solution (pH 1.2) for two hours to replicate gastric conditions. Subsequently, the pH was adjusted to 6.8 by adding concentrated phosphate buffer to simulate the intestinal environment. Each dissolution vessel was filled with 900 mL of medium, maintained at 37 °C, and stirred at 50 rpm. Samples of 1 mL were collected at scheduled time intervals over 36 h, filtered, and analyzed by HPLC (Section 3.3) to determine the cumulative percentage of drug released as a function of time.

### 3.7. Physicochemical Characterization of Zeolitic Materials

The physicochemical properties of natural mordenite (MR), zinc-modified mordenite (MR-ZN), drug-loaded mordenite with 10 and 23 mg of donepezil (DP-MR-ZN10 and DP-MR-ZN23), and formulations with microcrystalline cellulose (DP-MR-ZN10-CM and DP-MR-ZN23-CM) were comprehensively characterized. The morphology of the zeolitic materials was examined using Scanning Electron Microscopy (SEM), after sputter coating the samples with a thin layer of carbon. SEM analysis was conducted with a JEOL J-7100F microscope (JEOL Inc., Peabody, Massachusetts, MA, USA). Elemental composition was determined by X-ray fluorescence (XRF) using a Bruker model S1 Titan 800 handheld analyzer (Bruker, Billerica, MA, USA). Crystalline structure analysis was performed using a X-ray diffractometer (XRD, D8 Advance A25, Bruker, Karlsruhe, Germany), equipped with a Cu Kα anode radiation source (λ = 0.1542 nm), operating at 40 kV and 40 mA. Diffraction patterns were recorded in the 2θ range of 4° to 60°. Fourier Transform Infrared (FTIR) spectra were recorded in the range of 4000–500 cm^−1^ using a Nicolet iS10 spectrometer (Jasco 4100, Easton, MD, USA) operating at 50–60 Hz, after pressing the sample into a KBr pellet. The specific surface area was determined using a Micrometrics Chemisorb 2720 automatic adsorption analyzer (Norcross, GA, USA) by the single-point nitrogen adsorption method, employing a gas mixture of 30% N_2_ and 70% He. All measurements were performed at room temperature (22 ± 2 °C). The point of zero charge (PZC) of the natural and zinc-modified mordenite was determined using the pH drift method. For this purpose, 350 mg of zeolite were added to 10 mL of solution (H_2_O, and NaCl: 0.01 or 0.05 M) adjusted to initial pH values of 2, 4, 6, and 8 using HCl or NaOH. The suspensions were stirred for 24 h at room temperature, and the final pH was recorded. The PZC was identified as the point where ΔpH = 0.

### 3.8. Molecular Modeling and Density Functional Theory (DFT) Simulations

Density Functional Theory (DFT) simulations were performed according to methodologies described previously [73]. Calculations were carried out using the Vienna Ab Initio Simulation Package (VASP) version 6.0 (VASP Software GmbH, Vienna, Austria) [74,75]. Molecular modeling and visualization were conducted with BioVia Materials Studio version 5.5 (San Diego, CA, USA). Core and valence electron interactions were described using the projector augmented wave (PAW) method [76]. Electron exchange correlation functions were described using the Perdew-Burke-Ernzerhof (PBE) generalized gradient approximation (GGA). The selected cutoff energy for plane waves was set at 450 eV. The Kohn-Sham equations were iteratively solved until the total energy convergence criterion of 10^−5^ eV met [77]. The chosen dynamic configuration maintained the P1 point symmetry, with the Brillouin zone sampled via Monkhorst-Pack gratings centered at G [78]. The simulations were run under conditions without spin polarization, with atomic positions relaxed until the forces on them were reduced to less than 0.01 eV/Å. To assist in the convergence of the total energy, a Gaussian spot with a sigma value of 0.10 eV was applied. Starting from an orthorhombic unit cell, provided by Materials Studio software, the structural cell underwent full optimization, including initial cell shape and volume adjustments, followed by optimization of donepezil positions, zeolite models of isolated mordenite and the mordenite-donepezil combination. (MOR-DPZ) system. Using the MOR structure with a Si/Al ratio of 5 in a supercell configuration of 1 × 1 × 4 unit cells, the optimized lattice parameters of the orthorhombic cell were determined: a = 18.18, b = 20.37, c = 30.03 Å, with angles α = β = γ set at 90°, and the volume V was equal to 11,117 Å3. To synthesize the mordenite zeolite, 32 silicon atoms were replaced by 32 aluminum atoms, neutralizing the negative charge of each aluminum atom with a proton (H^+^), thus adding 32 protons to the structure. The distribution of aluminum atoms was conducted randomly, following Lowenstein’s rule [79]. Selecting the optimal configuration for the charge balance protons involved geometric optimizations to minimize the total energy. The resulting model consisted of 675 atoms, 802 bonds, and 192 polyhedra. To elucidate the chemical dynamics between donepezil and mordenite, a population analysis was conducted using the Bader approach, which offers an effective method to evaluate bond ionicity by quantifying the charge transfer between atoms [80]. Furthermore, charge difference analysis was employed to measure the charge redistribution in the mordenite structure caused by the adsorption of donepezil [20]. The electron localization function (ELF) also provided valuable information on the interaction characteristics between donepezil and mordenite [22], with the maximum electron density distribution (Region of Maximum Density or RMD) around the atomic nuclei indicating the potential for any of the ionic ones, according to Van der Waals, or covalent interactions, with covalent character inferred from the migration of RMDs between atomic centers toward a symmetrical arrangement [23]. Given the limitations of GGA functionals, especially in the description of long-range electronic correlations essential for van der Waals (vdW) forces, the incorporation of the Grimme dispersion correction method (DFT-D2) was considered necessary to simulate accurately the interactions within systems comprising large atoms. or molecules, including those with aromatic structures [81,82,83,84,85,86]. This correction was applied during simulations of both the mordenite structure and the adsorption processes of the donepezil molecule on the zeolite structure. Finally, adsorption energy of donepezil on mordenite zeolite was calculated using the following Equation (2) [87,88]:

∆E_ads_ = E_MOR-DON_ − E_MOR_ − E_DPZ_, [kJ mol^−1^] (2)

where

E_MOR-DPZ_: is the total energy of the zeolite-donenezil molecular supersystem.

E_MOR_: is the energy of the bare mordenite.

E_DPZ_: is the energy of the isolated donepezil molecule in vacuum.

## 4. Conclusions

This study demonstrates that zinc-modified mordenite (MR-ZN) is a promising carrier for the controlled release of donepezil, achieving high loading efficiencies (>93%) while preserving the crystallinity and structural integrity of the zeolite. The adsorption process followed a pseudo-second-order kinetic model (R^2^ > 0.99), indicating chemisorption as the predominant mechanism, complemented by secondary physisorption phenomena. This dual mechanism was further supported by intraparticle diffusion analysis, which revealed a multistage adsorption process dominated by surface interactions and gradual pore diffusion. Simulated gastrointestinal studies revealed a sustained, pH-dependent release profile, with 80% and 82% cumulative release after 24 h for the 10 mg and 23 mg formulations, respectively. These results confirm that the zinc-modified mordenite matrix provides a controlled and prolonged drug release, maintaining structural stability and minimizing burst effects during the initial stages of dissolution. Compared with conventional polymeric and mesoporous silica systems, the zinc-modified mordenite (MR–ZN) exhibits stronger drug–carrier interactions and sustained pH-responsive release. These properties enhance control over release kinetics and formulation robustness, positioning MR–ZN as a promising alternative for the controlled oral delivery of donepezil and similar therapeutic agents. DFT calculations confirmed the thermodynamic favorability of adsorption (−26.4 kJ/mol) and, together with Bader charge analysis and ELF mapping, identified hydrogen bonding, electrostatic forces, and van der Waals interactions as key contributors to drug–carrier stability and controlled desorption. It is important to note that the computational simulations were conducted using a pure mordenite framework; therefore, the potential contributions of minor mineral phases identified experimentally, such as clinoptilolite and montmorillonite, to the overall adsorption behavior are acknowledged as a limitation of the present theoretical approach. These aspects will be further explored in future studies through extended multi-phase modeling. Nevertheless, the integration of experimental and theoretical findings consistently supports the coexistence of both physical and chemical interactions, where coordination of Zn^2+^ with nitrogen and carbonyl groups of donepezil plays a central role in adsorption stability and release modulation. Overall, these findings highlight MR–ZN as a biocompatible, thermally stable, and cost-effective zeolitic platform for the design of advanced oral formulations. Future work should focus on in vivo pharmacokinetic and toxicity assessments, as well as zeolite surface functionalization strategies to fine-tune release kinetics, improve biocompatibility, and enhance clinical translation.

## Figures and Tables

**Table 1:** Cell parameters before and after full optimization.

Parameter	Mordenite (MOR)		Donepezil-Loaded Mordenite	
	Before	After	Before	After
a (Å)	18.09	18.17	18.18	18.18
b (Å)	20.52	20.17	20.37	20.37
c (Å)	30.10	29.84	30.03	30.03
α (°)	90.00	89.91	89.99	89.99
β (°)	90.00	91.59	91.12	91.12
γ (°)	90.00	91.49	90.85	90.54
V (Å^3^)	11,172	10,932	11,117	11,117

**Table 2:** Charge variation of atoms in the zeolite–donepezil complex based on Bader analysis.

Atoms	MOR-DPZ		
	BA (e)	AA (e)	ΔCharge
Si_MOR_	4.00	4.00	0.00
Al_MOR_	3.00	3.00	0.00
O_MOR_	−2.00	−2.00	0.00
H_MOR_	1.00	1.00	0.00
N_DON_	−2.38	−1.98	−0.40
C_DON_	0.26	0.26	0.00
O_DON_	−1.52	−1.62	+0.10
H_DON_	0.03	0.03	0.00

**Table 3:** Chemical composition of natural mordenite (MR) and zinc-modified mordenite (MR-ZN), loaded with 10 mg of donepezil (DP-MR-ZN10), mordenite loaded with 23 mg of donepezil (DP-MR-ZN23), mordenite loaded with 10 mg of donepezil and formulated with microcrystalline cellulose (DP-MR-ZN10-CM), and mordenite loaded with 23 mg of donepezil and formulated with microcrystalline cellulose (DP-MR-ZN10-CM).

Element	MR ± SD	MR-ZN ± SD	DP-MR-ZN10 ± SD	DP-MR-ZN23 ± SD	DP-MR-ZN10-CM ± SD	DP-MR-ZN23-CM ± SD
Al_2_O_3_ (%)	14.0 ± 0.9	15.3 ± 0.8	15 ± 0.9	14.5 ± 0.8	14.4 ± 0.8	13.6 ± 0.8
SiO_2_ (%)	63.2 ± 0.9	61.2 ± 0.8	63 ± 0.9	63.2 ± 0.8	64.6 ± 0.8	64.2 ± 0.8
S (%)	0.1 ± 0	0.1 ± 0	0.1 ± 0	0.1 ± 0	0.1 ± 0	0.1 ± 0
K_2_O (%)	0.7 ± 0	0.7 ± 0	0.7 ± 0	0.7 ± 0	0.7 ± 0	0.7 ± 0
CaO (%)	3.9 ± 0	3.5 ± 0	3.8 ± 0	3.9 ± 0	3.7 ± 0	3.7 ± 0
Fe_2_O_3_ (%)	2.5 ± 0	2.3 ± 0	2.4 ± 0	2.4 ± 0	2.3 ± 0	2.4 ± 0
ZnO (%)	15.6 ± 0	17.0 ± 0	15.1 ± 0	15.2 ± 0	14.3 ± 0	15.3 ± 0
Ti (%)	0.2 ± 0	0.1 ± 0	0.1 ± 0	0.1 ± 0	0.1 ± 0	0.2 ± 0
Mn (%)	0.1 ± 0	0.1 ± 0	<lq	<lq	<lq	<lq
Co (%)	<lq	<lq	<lq	<lq	<lq	<lq
Cu (%)	<lq	<lq	<lq	<lq	<lq	<lq
Ba (%)	0.1 ± 0.1	0.1 ± 0.1	<lq	0.1 ± 0	0.1 ± 0	<lq

SD: standard deviation; <lq: below the limit of quantification.

**Table 4:** Kinetic models and fitted parameters describing the adsorption behavior of donepezil on zinc-modified mordenite.

Model	Equation	Kinetic Parameters	
Pseudo-first order	$\ln\left(Q_{e}-Q_{t}\right)=\ln\left(Q_{e}\right)-K_{1}t$ where K_1_ (h^−1^) is the kinetic constant.	Q_e_ (mg∙g^−1^)	0.11
		K_1_ (h^−1^)	0.38
		R^2^	0.59
Pseudo-second order	$\frac{t}{Q_{t}}=\frac{1}{K_{2}Q_{e}^{2}}+\frac{t}{Q_{e}}$ where K_2_ (g·mg^−1^·h^−1^) is the kinetic constant.	Q_e_ (mg∙g^−1^)	5.58
		K_2_ (g∙mg^−1^∙h^−1^)	43.05
		R^2^	0.99
Intraparticular diffusion	qt=ktt12+A where Kt (mg·g^−1^·h^−1/2^) is the intraparticle diffusion rate constant and A (mg·g^−1^) is a constant that provides information about the thickness of the boundary layer.	Kt_1_ (mg∙g^−1^∙h^−1/2^)	64.61
		R^2^	0.92
		K_t2_ (mg∙g^−1^∙h^−1/2^)	0.03
		R^2^	0.99
		K_t3_ (mg∙g^−1^∙h^−1/2^)	0.05
		R^2^	0.90
		K_t4_ (mg∙g^−1^∙h^−1/2^)	0.01
		R^2^	0.91
Film Diffusion	$-\ln\left(1-\left(\frac{Q_{t}}{Q_{e}}\right)\right)=\frac{D_{F}c_{s}}{hrc_{z}}t$ where D_F_ (m^2^∙h^−1^) is the diffusion coefficient, c_s_ (mg·L^−1^) and c_z_ (mg·kg^−1^) are donepezil concentrations in solution and in the adsorbent, respectively, r is the average radius of the zeolite particle (particles below 200 mesh ≈ radius: 3.7 × 10^−5^ m), t is the contact time (min) and h is the film thickness of the adsorbent particle (1 × 10^−5^ m for a poorly stirred solution).	D_F_ (m^2^∙h^−1^)	4.3 × 10^−9^
		R^2^	0.66
Particle Diffusion	$-\ln\left(1-{\left(\frac{Q_{t}}{Q_{e}}\right)}^{2}\right)=\frac{2\pi^{2}D_{P}}{r^{2}}t$ where D_P_ (m^2^∙h^−1^) is the diffusion coefficient	D_P_ (m^2^∙h^−1^)	2.9 × 10^−11^
		R^2^	0.72

## Data Availability

The data presented in this study are available in this article.

## Supplementary Materials

The following supporting information can be downloaded at: https://www.mdpi.com/article/10.3390/molecules30214174/s1, Figure S1: Speciation diagram of Zn^2+^ in acetate medium as a function of pH; Figure S2. Predicted pH-speciation diagram of donepezil obtained from ChemAxon pKa calculator.
