# Supplementary material for: Zinc-Modified Mordenite Zeolite as a Molecular Carrier for Donepezil: A Framework for Drug Delivery Applications
Source: Molecules. 2025 Oct 24;30(21):4174. doi: 10.3390/molecules30214174 (PMC12610327; doi:10.3390/molecules30214174)
Supplement: Supplementary file 1 [file molecules-30-04174-s001.zip › molecules-3885781-supplementary.pdf]

Article

Zinc-Modified Mordenite Zeolite as a Molecular Carrier for Donepezil: A Framework for Drug Delivery Applications

**Diana Guaya [1], Lupe Carolina Espinoza [1,2,*], Ximena Jaramillo-Fierro [1], Dagmar Gualotuña Campoverde [3],
Lilian Sosa [4,5] and Ana Cristina Calpena [6,*]**

[1] Departament of Chemistry, Universidad Técnica Particular de Loja, Loja 1101608, Ecuador;
deguaya@utpl.edu.ec (D.G.); xvjaramillo@utpl.edu.ec (X.J.-F.);

[2] Institute of Nanoscience and Nanotechnology (IN2UB), University of Barcelona, 08028 Barcelona, Spain

[3] Biochemistry and Pharmacy Degree, Universidad Técnica Particular de Loja, Loja 1101608, Ecuador; degualotuna1@utpl.edu.ec (D.G.C.)

[4] Institute of Microbiology Research (IIM), Faculty of Sciences, National Autonomous University of
Honduras (UNAH), Tegucigalpa 11101, Honduras; lilian.sosa@unah.edu.hn

[5] Pharmaceutical Technology Research Group, Faculty of Chemical Sciences and Pharmacy, National
Autonomous University of Honduras (UNAH), Tegucigalpa 11101, Honduras

[6] Department of Pharmacy, Pharmaceutical Technology and Physical Chemistry, Faculty of Pharmacy and Food Sciences, University of Barcelona, 08028 Barcelona, Spain

* Correspondence: lcespinoza@utpl.edu.ec (L.C.E.); anacalpena@ub.edu (A.C.C.)

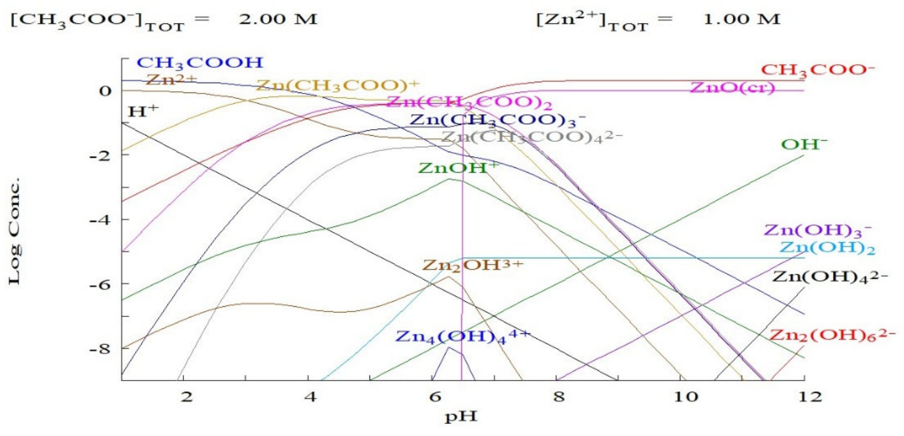

Figure S1. Speciation diagram of Zn^{2+} in acetate medium as a function of pH.

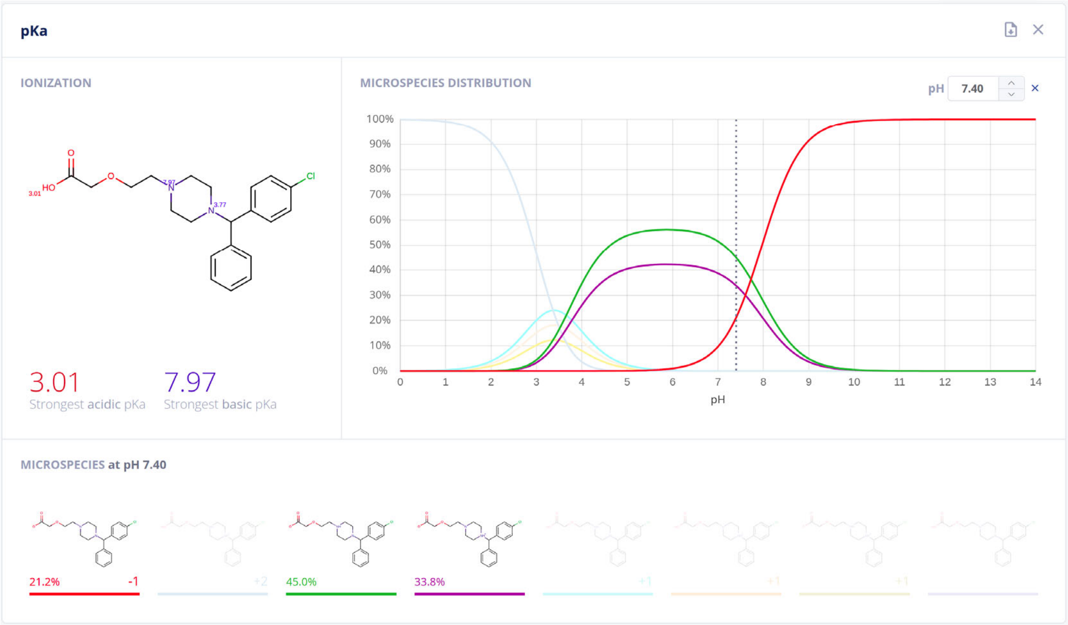

Figure S2. Predicted pH-speciation diagram of donepezil obtained from ChemAxon pKa calculator (https://chemaxon.com/calculators-and-predictors).
